# A Case of an Obstructive Intramural Haematoma during Percutaneous Coronary Intervention Successfully Treated with Intima Microfenestrations Utilising a Cutting Balloon Inflation Technique

**DOI:** 10.1155/2018/4875041

**Published:** 2018-11-19

**Authors:** Osama Alsanjari, Aung Myat, James Cockburn, Grigoris V. Karamasis, David Hildick-Smith, Andreas S. Kalogeropoulos

**Affiliations:** ^1^Department of Cardiology, Royal Sussex County Hospital, Brighton and Sussex University Hospitals NHS Trust, Brighton, UK; ^2^Department of Cardiology, The Essex Cardiothoracic Centre, Basildon, UK

## Abstract

During percutaneous coronary interventions (PCI), good lesion preparation with adequate balloon predilatation is a fundamental step before stent deployment in order to achieve optimal stent expansion and favourable long-term outcomes post PCI. During PCI, inadvertent vessel tearing can occur, resulting in coronary dissections and formation of intramural haematomas. The latter might be associated with compression of the vessel lumen and significant compromise of the coronary blood flow leading to myocardial ischaemia and infarction. Herein, we present a case of intramural haematoma that occurred after PCI of the left anterior descending artery resulting in occlusion of the vessel and the subsequent use of a cutting balloon inflation technique to resolve the haematoma and restore the normal coronary blood flow.

## 1. Introduction

Appropriate lumen enlargement with balloon predilatation is an essential part of successful stent deployment and adequate expansion during percutaneous coronary interventions (PCI) [1, 2]. Plaque fracture with dissection of the arterial wall after balloon predilatation is considered a key component of this process. However, during subsequent stent deployment, inadvertent vessel tearing may also occur at the transition point between the rigid stent struts and the adjacent arterial wall [3]. This injury can have various manifestations from innocuous small edge dissections to intramural haematomas. The latter form is defined as blood accumulation in the medial space with displacement of the internal elastic membrane inward and the external elastic membrane outward with or without identifiable entry or exit point [4]. Post-PCI intramural haematomas can cause extensive compression of the true lumen of the vessel, resulting in myocardial ischaemia and myocardial infarction [5]. Bailout treatment of intramural haematomas through further stenting, reentry with a stiff chronic total occlusion- (CTO-) dedicated guidewire, or microfenestrations with cutting or scoring balloons has been previously described [6–12]. However, there is still no consensus regarding the optimal treatment strategy. Herein, we present a case of a flow-limiting intramural coronary haematoma following PCI that was successfully treated with microfenestrations of the intima layer, utilising a cutting balloon inflation technique.

## 2. Case Report

A 70-year-old man transferred to our institution with a 24-hour history of intermittent central chest pain and evidence of inferior ST elevation myocardial infarction (STEMI) on the twelve-lead electrocardiogram. He was an ex-smoker with a history of 10 pack-years without any significant past medical history and not on any regular medications.

**Figure 1:**
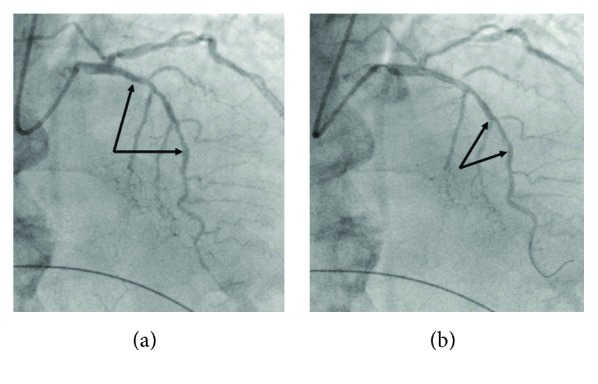
(a) Long segment of moderate to severe stenosis in the LAD. Arrows indicate the target segments for deployment of the stents. (b) Intima flap, following lesion predilatation (arrows).

Given his symptoms and the associated ECG findings, he was transferred to the catheterisation laboratory and an emergency coronary angiography was performed via the right radial artery access route using a 6Fr arterial sheath. The left main (LMS) and the right coronary artery (RCA) were engaged with a 5f Judkins left (JL) 3.5 diagnostic catheter and a 6f Judkins right (JR) 4.0 guiding catheter, respectively. The left coronary system revealed a patent LMS, a tubular moderate to severe stenosis extending from the proximal to the mid segment of the left anterior descending artery (LAD) (Figure 1(a)), and a minor nonobstructive atheroma in a nondominant left circumflex artery (LCx). The RCA was acutely occluded. We proceeded to primary PCI of the RCA. Using the 6Fr JR4 guiding catheter, a Sion Blue guide wire (Asahi INTECC Co., Ltd.) was passed through the occluded segment into the distal vessel. Predilatation was then performed with a 2.5 mm Trek, semicompliant, balloon (Abbott Vascular) with immediate restoration of TIMI 3 flow. This revealed a critical mid vessel stenosis, which was subsequently stented with an Orsiro (BIOTRONIK) 3.5 × 30 mm drug eluting stent (DES), deployed at 12 atmospheres and postdilated with a 3.75 × 20 mm Accuforce (Terumo) noncompliant (NC) balloon inflated to 20 atmospheres with an excellent final angiographic result.

**Figure 2:**
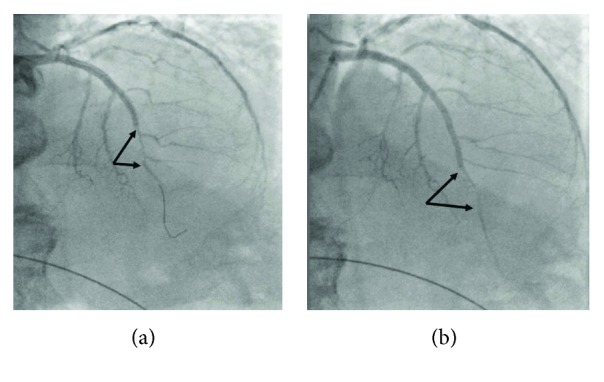
(a) Significant luminal stenosis at the level of two small diagonal branches (arrows) after deployment of the stents and postdilatation of the overlapping segment with the 3.5 mm stent balloon at 16 atmospheres. Stenosis did not resolve after intracoronary administration of two consecutive 300 mcg bolus doses of nitrates. (b) Further overlapping stenting resulted in propagation of the haematoma and significant distal luminal stenosis after the level of the two small diagonal branches (arrows).

**Figure 3:**
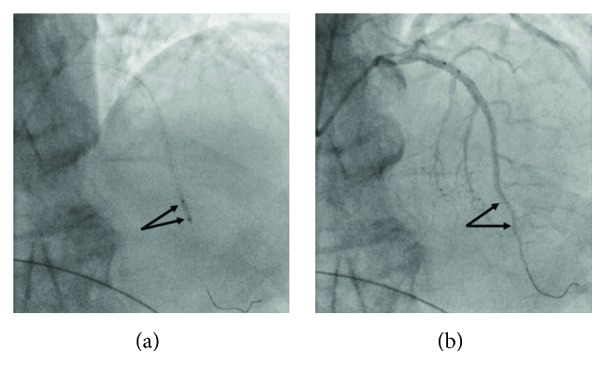
(a) Inflation of a 2.5 mm cutting balloon at nominal pressure at the transition of the stent struts to the adjacent unstented reference segment (arrows). (b) Restoration of normal TIMI 3 flow after inflation of cutting balloon at nominal pressure (arrows).

In light of the significant bystander disease, we elected to treat the proximal-mid LAD stenosis during the index procedure. Using a 6Fr EBU 3.5 guiding catheter, the lesion was crossed with a Sion Blue guide wire and predilatation was performed with a Trek (Abbott Vascular), 2.5 × 20 mm, semicompliant balloon followed by a 3.0 × 20 mm Accuforce (Terumo) NC balloon at 12 atmospheres. Following predilatation, we could easily identify an intima flap within the stenotic lesion (Figure 1(b)). Aiming to cover the dissection flap, we deployed a 2.75 × 26 mm Orsiro (BIOTRONIK) DES at the mid segment, which was overlapped proximally with a 3.5 × 20 mm Orsiro (BIOTRONIK) DES. The overlapping part of the stents was then postdilated with the 3.5 mm stent balloon at 16 atmospheres. The succeeding cine-acquisition demonstrated a significant luminal stenosis at the outflow of the distal stent (Figure 2(a)), associated with new dynamic anterior ST elevation on the ECG and chest discomfort of the patient. The administration of two consecutive bolus 300 mcg of intracoronary nitrates failed to resolve stenosis and restore normal blood flow, a finding in keeping more likely with a plausible iatrogenic intramural haematoma rather than a coronary spasm. As the patient was clinically unstable and rapidly deteriorating, we initially opted not to perform further assessment with an intravascular ultrasound (IVUS) and quickly treat the haematoma with an additional stent implantation, but after deploying a 2.5 × 18 mm Orsiro (BIOTRONIK) DES, the subintimal haematoma was propagated more distally with further subtotal occlusion of the vessel (Figure 2(b)). At this stage, we selected to treat the haematoma with a cutting balloon inflation technique, intending to create multiple microfenestrations and relieve the excess inner luminal pressure caused by the haematoma. First, a 2.5 × 6 mm Flextome (Boston Scientific) cutting balloon was successfully delivered at the outflow of the distal stent. Then, we inflated the balloon at nominal pressure, exactly at the transition point between the distal edge of the stent and the adjacent unstented vessel wall (Figure 3(a)). Successive angiography demonstrated complete resolution of the luminal stenosis with complete restoration of TIMI 3 flow (Figure 3(b)). The patient's troponin level from a blood sample taken on admission was 4528.00 ng/L (normal range 0–14 ng/L), and an echocardiogram two days after his index procedure showed preserved left ventricular systolic function with an ejection fraction of 55–60% and regional wall motion abnormalities involving hypokinesia of the basal to mid-inferior and inferolateral wall segments. The rest of the myocardial segments had normal contractility. The patient had further follow-up in the outpatient cardiology clinic 3 months later, where he reported an excellent recovery and denied any recurrent symptoms. Additional follow-up has been scheduled for one year after his successful PCI and myocardial infarction.

## 3. Discussion

Subintimal haematoma is a well-recognised complication after PCI. It is defined as blood accumulation in the medial space with displacement of the internal elastic membrane inward and the external elastic membrane outward with or without distinguishable entry or exit points, and its diagnosis is confirmed with the utilisation of intravascular imaging modalities, such as the IVUS and optical coherence tomography (OCT) [4]. A previous observational IVUS study, in an unrestricted cohort of patients including 1025 subjects, demonstrated an overall incidence of 6.7%. Furthermore, 60% of the detected haematomas had an angiographic appearance of an intimal dissection flap, and in 29% of those, no significant angiographic abnormality was identified [5]. The underlying mechanism of dissection and subsequent intramural haematoma is multifaceted. Previous pathological findings indicate that an intramural haematoma begins as a dissection to the media and propagates along the medial plane into more normal arterial segments but does not reenter the lumen. After stent implantation, the dissection usually occurs at the edge of the stent, at the transition point between the rigid struts and the nearby unstented reference segment. Once medial dissection occurs, haematoma formation and expansion seem to require a normal arc of arterial wall, whilst a scarred diseased media behind an atherosclerotic plaque area may prevent the propagation of the dissection [5]. A previous observational study of post-PCI edge dissections involving OCT morphometric analysis has shown that a stent landing zone involving an area with significant plaque burden can increase the risk of stent edge dissection by more than 6 times. In addition, circumferential calcium angle, fibrous cap thickness, and vessel overstretching were also independent predictors for the occurrence of edge dissections [13]. In this study, 10% of the edge dissections were associated with the formation of subintimal haematoma [13].

Although it is a rare PCI complication, intramural haematoma can be associated with a pressure-driven enlargement of the medial space with vessel lumen compression resulting in significant blood flow disturbances and ischaemia. Prompt recognition and immediate treatment in this context are paramount in order to restore the normal coronary blood flow and resolve ischaemia. As it may prove challenging to recognise the haematoma by angiography alone, intravascular imaging with OCT and IVUS can help to delineate the relevant anatomy and confirm the diagnosis [14]. By IVUS, an intramural haematoma appears as a homogeneous, hyperechoic, crescent-shaped area. The echogenicity of the blood depends on the flow rate, red cell aggregation, and fibrin contents [5]. In the OCT, the intramural haematoma appears as a low-signal area with low light attenuation usually located at the level of the media tunica and demarcated externally by the adventitia [13]. However, forceful injection of contrast media that usually encompasses OCT imaging can increase the risk of further expansion of the medial dissection with more distal propagation of the haematoma. In our case, we opted not to perform an IVUS or OCT as the patient was rapidly deteriorated with profound symptoms of chest pain and dynamic ECG changes. In addition, the constellation of angiographic findings and the ineffective administration of intracoronary nitrates were highly suggestive of intramural haematoma diagnosis.

**Figure 4:**
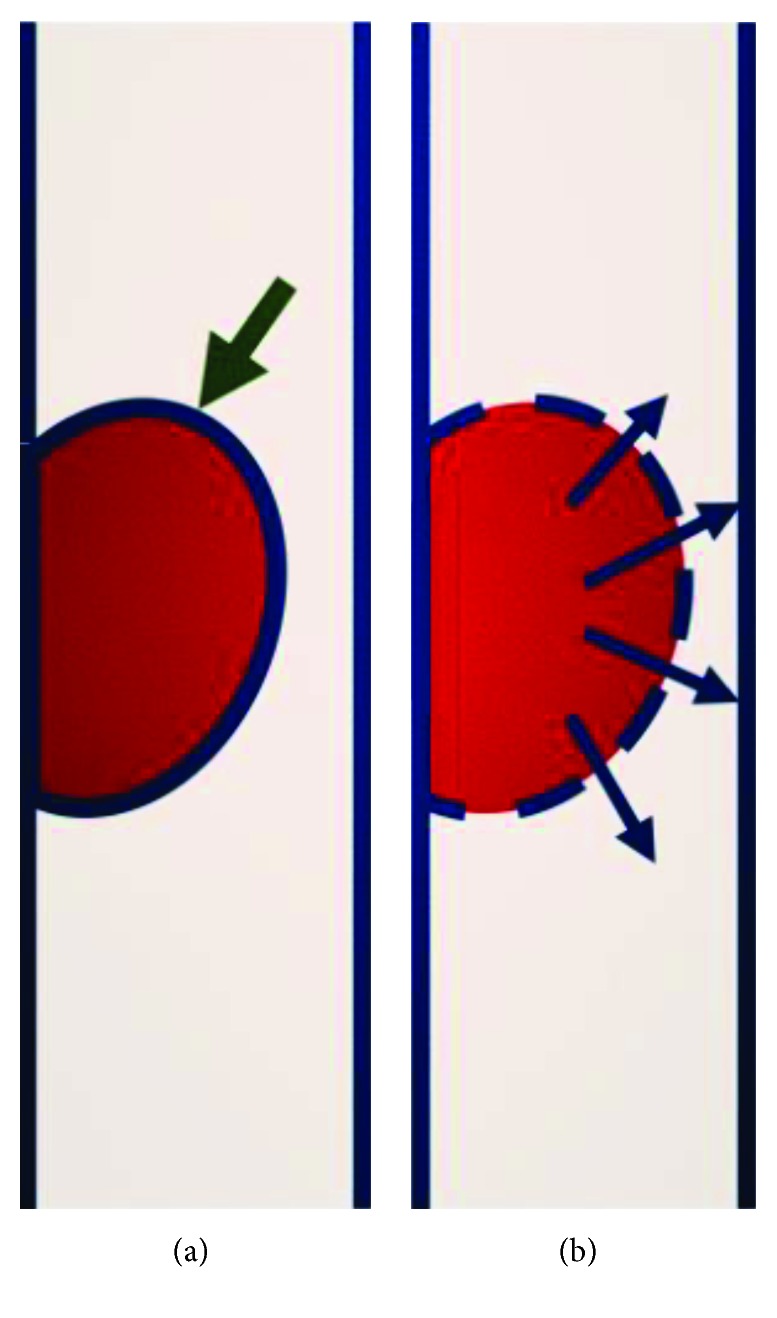
(a) Intramural haematoma (green arrow) can cause luminal obstruction, with significant flow disturbances resulting in significant ischaemia and ensuing myocardial infarction. (b) After single cutting balloon inflation at nominal pressure, the balloon blades generate several microfenestrations on the intima surface. The result is the creation of various blood exit points (blue arrows) that relieve the internal haematoma pressure towards the lumen of the vessel and restore normal blood flow.

So far, the management of post-PCI intramural haematoma remains controversial and there is no general consensus on optimal therapeutic strategy. Previously published reports have highlighted the role of further stenting, reentry with a stiff CTO guide wire, and also intimal fenestrations with either a cutting or a scoring balloon [6–12]. Treatment with an additional stent embraces an additional risk of further propagating the haematoma more distally, as this was evidently shown in our case. In addition, the use of a stiff guide wire might prove difficult as the manipulation of the wire usually requires expert skills and might also be time consuming. In our case, we applied an elegant and practical technique to treat the haematoma involving the creation of intima microfenestrations by using a cutting balloon. We chose a balloon-artery size ratio of 1 : 1 and specifically aimed to inflate the balloon at the site of the distal edge of the stent and only on nominal pressure to minimise the extent and degree of arterial wall injury. The rationale of this technique is that the blades of the balloon would easily create microfenestrations and reentry points at the intima that will eventually allow the intramural blood to exit and induce decompression of the medial space with successive resolution of the luminal stenosis (Figure 4). In addition, minimal vessel trauma will ensure smooth healing of the vessel wall without the need for further stent deployment and increased odds for optimal long-term outcomes without major adverse coronary events. In a previous case report of a spontaneous left main coronary artery dissection, treatment with stents resulted in propagation of the intramural haematoma with subsequent compromise of coronary blood flow that was successfully dealt with a cutting balloon inflation, subsequent resolution of the haematoma, and restoration of coronary blood flow. The patient had an excellent recovery and remained asymptomatic at one-year follow-up [12]. Similarly, in two cases with a CTO, PCI using an antegrade and retrograde dissection and reentry technique stent deployment resulted in a compressive intramural haematoma associated with coronary flow compromise. In both occasions, the haematomas were resolved with a cutting balloon inflation and the patients remained asymptomatic without any adverse events at one-year follow-up [11]. In another report, where a balloon predilatation resulted in extensive vessel dissection, a compressive blood flow-limiting haematoma ensued stent deployment. This was successfully treated with a scoring balloon inflation and reinstatement of normal coronary blood flow. Cardiac biomarkers remained within normal range at 6 and 12 hours after procedure. In addition, follow-up coronary angiograms at three months and one year demonstrated an occluded false lumen and a normal coronary flow at the distal vessel [8].

In our case, the patient presented with an acute ST elevation myocardial infarction, and therefore, we performed a single measurement of troponin levels on admission. Even though after inflation and retraction of the cutting balloon the distal vessel lumen diameter appeared slightly smaller compared to the initial angiogram before the PCI, this is an expected finding secondary to distal vasomotion and associated coronary spasm that have been induced by the vessel injury. Moreover, the specific intervention successfully restored a normal TIMI 3 flow and the patient remained completely asymptomatic at three months after his hospital discharge with a plan for further follow-up at one year after his PCI.

## 4. Conclusion

Intramural haematoma is an uncommon complication of PCI, which can often be associated with detrimental clinical fallouts including profound ischaemia. Prompt recognition and treatment are of paramount importance. The utilisation of a cutting balloon technique, aiming to create microfenestrations of the intima layer of the arterial wall, is a simple, elegant, and practical method to effectively treat iatrogenic intramural haematoma after stent deployment that has been associated with excellent short and midterm outcomes.
